# Patterns of cytotoxic T-cell densities in immunogenic endometrial cancers reveal a potential mechanism for differences in immunotherapy efficacy

**DOI:** 10.1136/bmjonc-2024-000320

**Published:** 2024-05-21

**Authors:** Neil Ryan, Mark Glaire, Thomas Walker, Natalja ter Haar, Marieke Ijsselsteijn, James Bolton, Noel de Miranda, Gareth Evans, David N Church, Tjalling Bosse, Emma Crosbie

**Affiliations:** 1 The University of Manchester, Manchester, UK; 2 The University of Edinburgh, Edinburgh, UK; 3 Department of Gynaecology Oncology, Royal Infirmary of Edinburgh, Edinburgh, UK; 4 University of Oxford, Oxford, UK; 5 Lancaster University, Lancaster, UK; 6 Department of Pathology, Leiden University Medical Centre, Leiden, The Netherlands; 7 Department of Histopathology, Manchester University NHS Foundation Trust, Manchester, UK; 8 Manchester Academic Health Science Centre, Manchester, UK; 9 Central and South Genomic Medicine Service Alliance, Oxford, UK

**Keywords:** Lymphocytes, Endometrial cancer, Immunotherapy

## Abstract

**Objective:**

To explore the impact of molecular subtype in endometrial cancer (EC) on CD8+T cell densities. Furthermore, this work will test the assumption that all mismatch repair deficient (MMRd) tumours are immunologically similar which would enable current trial data to be generalised to all MMRd ECs.

**Methods and analysis:**

All tumours were characterised into the four clinical molecular subtypes. For analysis, the *TP53* mutant and no-specific molecular profile tumours were grouped together and described as the low mutational burden (LMB) cohort. CD8+T cell counts were taken from four regions of interest which sampled the tumour-stromal interface and the tumour core. CD8+T cell counts were analysed as mean averages.

**Results:**

In total, 607 ECs contributed to the analysis. CD8+T cell counts in confirmed Lynch syndrome (LS) ECs were significantly higher than *MLH1*-methylated ECs in all tumour locations excluding the tumour stroma. Confirmed LS and path_*POLE* ECs had significantly higher CD8+T cell counts across all tumour locations when compared with LMB ECs. There were limited significant differences in CD8+T cell counts between path_*POLE* versus confirmed LS ECs. There was no significant difference in the CD8+T cells counts and gene (*MLH1*, *MSH2*, *MSH6*, *PMS2*) in which the LS pathogenic variant was found; however, this analysis was limited by small numbers.

**Conclusion:**

These data indicate that CD8+T cell numbers and distribution is not equal between *MLH1*-methylated and confirmed LS ECs. This is relevant when interpreting current trial data looking to the application of checkpoint inhibition treatments in MMRd cancers.

**WHAT IS ALREADY KNOWN ON THIS TOPIC**

- Immunotherapy has proved high successful in mismatch repair deficient (MMRd) endometrial cancer (EC). However, very little is known about why immunotherapy fails in certain patients with MMRd or about how it could be applied to other molecular subgroups.
- Current treatment studies treat all MMRd EC as a homogeneous eternity despite there being distinct aetiologies to MMRd.
- A comprehensive description of CD8+T cell populations in different molecular subgroups of EC is required to better understand treatment variation and potential.

**WHAT THIS STUDY ADDS**

- CD8+T cell populations are different between molecular subgroups and between sporadic and hereditary MMRd ECs.
- Lynch syndrome (LS) associated ECs and path_*POLE* have the highest CD8+T populations. Sporadic *MLH1*-hypermethylated ECs are also immunogenic but to a lesser degree.

**HOW THIS STUDY MIGHT AFFECT RESEARCH, PRACTICE OR POLICY**

- These data will inform those looking to better understand immunotherapy treatment variation in ECs.
- Furthermore, those wishing to undertake trials in immunotherapy should attempt subgroup analysis on treatment efficacy stratified by the aetiology of MMRd and not seek to treat MMRd EC as a homogenous group.
- Although a prognostically favourable group, path_*POLE* EC shares immunological similarities with MMRd EC and therefore could be amenable to immunotherapy; this warrants further study.

## Introduction

 Endometrial cancer (EC) is the most common gynaecological malignancy in the Western world; the incidence of EC continues to rise globally year-on-year, primarily due to rising obesity levels and an ageing population.^1^ With an increasing incidence and an associated rise in recurrent disease, more women are dying of EC cancer than at any other time.^2^ This is further complicated by an increasing degree of complexity seen within EC patients, with far higher levels of class III obesity related sequelae and a lower performance status, making surgical treatment problematic.^3^

Historically, the treatment of EC was directed by histological features such as grade and stage of disease.^2^ Recently there has been movement towards a personalised molecular approach, with the recent literature looking to the genomic landscape of the disease to inform clinical management.^4^,^6^ Since the publication of The Cancer Genome Atlas’ report on EC, the molecular aetiology of the disease has been viewed in four distinct groups: *POLE* mutated (both somatic and germline), mismatch repair deficient (MMRd) (both somatic and germline), copy number high and copy number low variants.^7^ These groups are distinct both in their biology but also in their clinical outcome, a finding that has been ratified in independent cohorts.^8 9^ Therefore, the identification and incorporation of molecular classification is of prognostic importance and helps direct treatment, especially the use of immunotherapy.^10^

There is growing evidence that distinct molecular entities in EC illicit different immune responses.^11 12^ EC that arise from a defective mismatch repair system (MMR), either through germline or somatic pathogenic variants in *MLH1*, MHS2(*EPCAM*), *MSH6*, *PMS2* or their epigenetic controls (such as hypermethylation of the *MLH1* promotor region, which is by far the most frequent) are known to have a high number of tumour infiltrating lymphocytes (TILs).^11^ Furthermore, there are differences in the degree of numbers of TILs dependent on the mechanism of MMRd, be it somatic or as a result of Lynch syndrome (LS); an autosomal dominant inherited pathogenic mutation affecting one of the MMR genes.^13 14^ ECs with mutations in the DNA Polymerase Epsilon, Catalytic Subunit (path_*POLE*) are also more immunogenic then those without a path_*POLE*.^15 16^ Immune profiling solid tumours also has a prognostic function, with CD8+T cell derived ‘immune-scores’ being highly predictive of clinical outcome.^13 14 17^ Furthermore, it has implications for treatment.

The immune response in EC with a high mutational burden is thought to derive from the production of immune active frame-shift proteins that act as neo-antigens.^18^ This in turn applies a selective pressure on the tumour, encouraging immune-editing and ultimately immune escape often mediated through the PD-1/PD-L1 axis.^19^ This is a druggable pathway and pharmacological treatments blocking the axis have shown promise in MMRd ECs.^20 21^ However, these studies had an overrepresentation of LS tumours (up to 48%) which does not reflect the clinical situation whereby the vast majority of MMRd tumours arise through somatic events.^22^ It is therefore important to explore the immune landscape of MMRd tumours by their aetiology (somatic vs inherited) to ensure they are biologically comparable and composite treatment predictions are meaningful. In addition, not all MMRd ECs respond to immune therapy; therefore, it is important to establish a detailed picture of the immune landscape within these cancers to see if variance could help explain the clinically observed treatment heterogeneity.^14 21^

The aim of this study was to explore the density of CD8+T TILs in different molecular groups of EC. Furthermore, by describing these densities by tumour compartment we aim to explore the underlying immune environment of these tumours. By using a potentially fully automatable processes we looked at the feasibility of generating automated CD8+T cell counts in a way that would have minimal implications for clinical workflows. Overall, we believe these results will inform the optimisation of immunotherapy in EC.

## Materials and methods

### Patients and tissue

#### Patient and public involvement statement

This work was supported by LS UK, an all-volunteer organisation founded and governed by LS survivors and their families. Members of LS UK agreed to donate samples to this study and agreed that it was a research priority for patients. Furthermore, members of LS UK were consulted on the set up of this study and have been kept informed about the progress and outputs of this work.

More generally, the objectives of this study are viewed as important by EC survivors. A patient derived top 10 unanswered research questions in EC, ranked the efficacy of treatments for advanced EC based on molecular pathways as number three.^23^

#### Tissue

ECs were sourced from the Biomarkers of Lynch Tumours study (BOLT) and the Post-Operative Radiation Therapy in Endometrial Carcinoma 1 and 2 (PORTEC 1 and 2) trials. The recruitment and selection criteria are outlined in detail for the PORTEC and BOLT studies in the literature.^14 24 25^ Pathology in the PORTEC cohort was contemporaneously confirmed by an experienced gynaecology specialised histopathologist (TB). All women recruited to BOLT provided a clinical genetics report as evidence of their LS diagnosis. For BOLT, contemporaneous histology review was completed by an experienced gynaecology specialised histopathologist (JB). Tumours were then sectioned into 4 µm thick slices and mounted on electrostatic adhesive slides (Thermo Fisher Scientific, Waltham, MA, USA) by the research team.

### Molecular classification

For the PORTEC derived samples MMR protein expression, microsatellite instability (MSI), *MLH1*-hypermethylation and somatic path_*POLE* was determined a priori as previously described.^8^ Those with copy number high or copy number low profiles were assigned to low mutational burden (LMB) group. In addition, the suspected LS cohort was defined as tumours that showed loss of either MLH1, MSH2, MSH6 or PMS2, individually or in their dimeric couples, and did not display hypermethylation of *MLH1* and had not undergone germline LS testing. This ‘Lynch Like’ group is important as many healthcare settings do not have access to routine germline sequencing.

For the BOLT derived samples, all patients had clinically confirmed germline path_*MMR* pathogenic variants. In addition, the absence of the corresponding path_*MMR* gene protein expression was confirmed in the tumour through immunohistochemistry (IHC). This has also been described in the literature.^26 27^ Briefly, the MMR IHC was carried out according to standardised clinical protocols. MLH1, MSH2, MSH6 and PMS2 staining was performed using the automated IHC platform Ventana BenchMark ULTRA IHC/ISH Staining Module (Ventana, Tucson, AZ, USA) together with the OptiView, 3,3′ diaminobenzidine version 5 detection system (Ventana) on 4 µm formalin fixed paraffin fixed freshly sectioned slides. Scoring was performed independently by two experienced scorers (NR and TB); scoring disagreements were resolved by conference scoring with an experienced gynaecological specialised pathologist (TB) having the deciding vote.

### Immunofluorescence

Characterisation of CD8+T cells was carried out using duplex immunofluorescence. Tissue sections were deparaffinised in xylene and rehydrated in sequential ethanol concentrations. Endogenous peroxidase was blocked by incubation in a 0.3% hydrogen peroxide/methanol (Merck Millipore, Burlington, MA, USA) solution for 30 min. This was followed by antiretrieval in a heated Tris-EDTA (10 mM/1 mM, pH 9) solution which was then cooled to room temperature. Non-specific antibody binding was achieved with 30 min of incubation with 10% normal goat serum (Dako). Slides were then washed in phosphate-buffered saline (PBS) and were incubated overnight with the primary antibodies in a dark box. Slides were again washed in PBS and incubated for 1 hour with a solution of their fluorescent secondary antibodies diluted in PBS/Bovine serum antigen 1%. All antibodies are detailed in online supplemental table S1. Slides were washed again in PBS and air dried. Finally, slides were incubated with DAPI (1 µM) as nuclear counterstain and mounted with Prolong Gold Antifade Reagent (Cell Signaling Technology, Danvers, MA, USA). Slides were then dried at 30°C overnight before being stored in a cold room in dark conditions. Tonsil acted as a positive control for each run whereas the negative control was performed on EC tissue that underwent all stages bar the addition of the primary antibodies.

### Image acquisition and analysis

**Figure 1:**
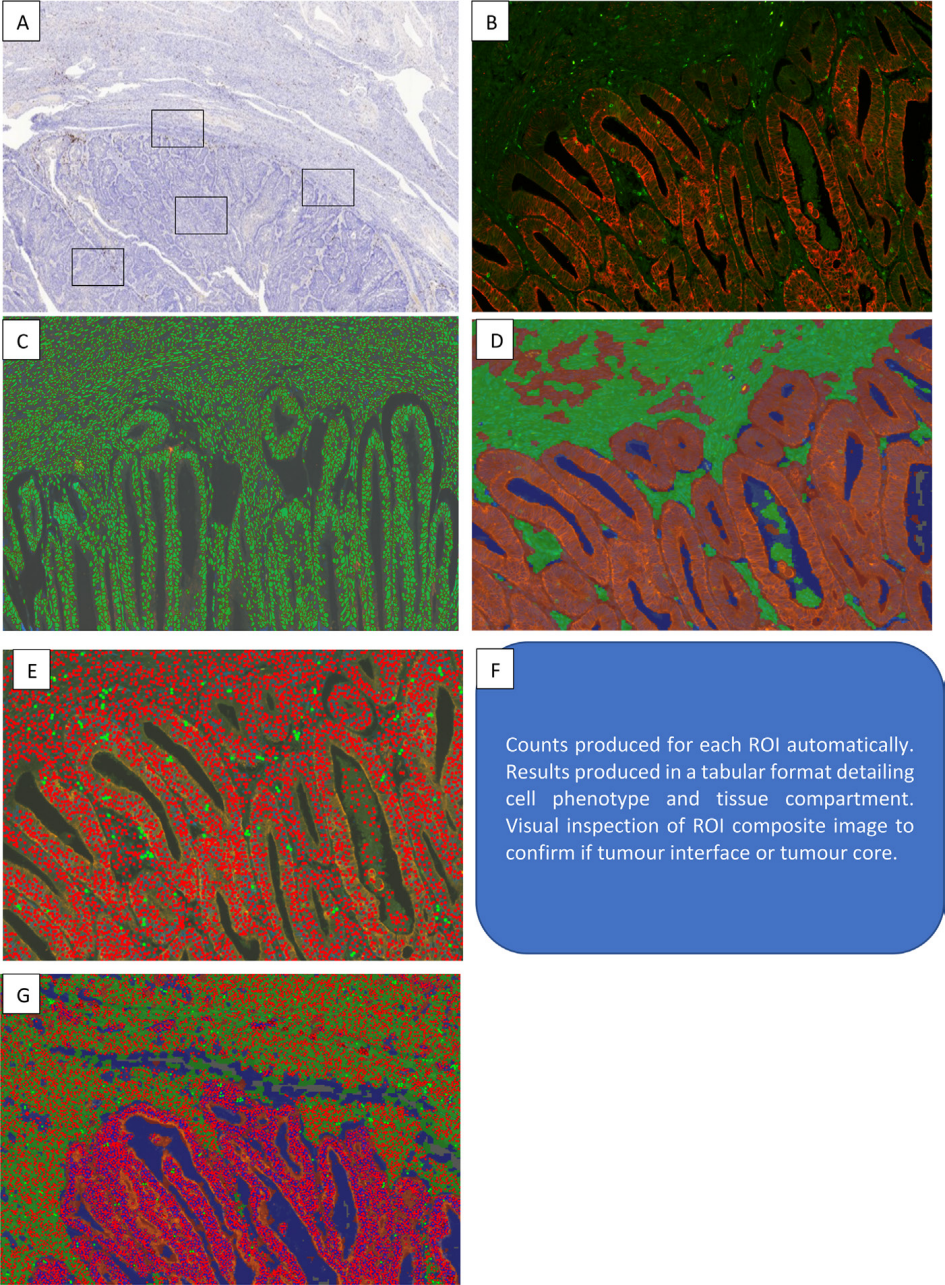
Workflow for image preparation and analysis. (**A**) Whole slide scans reviewed for quality and four regions of interest (ROI) selected manually for high resolution scan. (**B**) ROI individually visually inspected. Cell contouring (**C**), tissue segregation (**D**), cell phenotyping solutions run (**E**) generating cell phenotype counts by tissue segmentation (**F**). These data are exported to spreadsheet format. Composite images quality assured manually against ROI to assure accuracy. If errors identified, manual count conducted.

All slides were scanned within 14 days of immunofluorescence staining. They were imaged using the Vectra 3.0 Automated Quantitative Pathology Imaging System (Perkin Elmer) at 40× magnification as per manufacturer’s instructions. Image analysis and spectral separation was performed using InForm Cell Analysis Software (Perkin Elmer). Spectral separation was informed by the single marker spectral libraries and optimised by eye. Image analysis workflows were then created for high volume analysis. This was achieved manually with DAPI and keratin to segment images into tumour, stroma, necrosis and ‘no tissue’ areas. Cell segregation was optimised with the use of the DAPI stain to detect cell contours. Cell phenotype (CD8+T or CD8−) was trained in 40 tumours with 40 cells per tumour (see figure 1).

For each tumour, four regions of interest (ROI) were manually selected covering an area of 20 μm×20 μm. Two ROI were selected (by NR) at the tumour/stromal interface and two covered the tumour core. In those cases where there was no tumour/stromal interface (such as endometrial biopsies) only two ROI’s were taken within the tumour core. Chromogenic histology was visually checked to ensure accurate tissue segregation and CD8+/−T cell identification for each ROI; in cases of inaccurate counts a manual count was performed (see online supplemental figure S1). If imaging or segregation/CD8+T cell fluorescence failed, preventing manual counting, the ROI was excluded from analysis.

### Statistical analysis

Samples were anonymised as to blind those involved in the analysis as to the molecular status of the tumours. Data tidying and consolidation was conducted by VBA scripting and conditional formulae in Microsoft Excel 2010. Statistical analysis was conducted in R 3.4.0×64 called from RStudio IDE 1.0.143 under Windows 7×64 and GraphPad PRISM (V.7 La Jolla California USA). Data for sample CD8+T cell counts and molecular profile was integrated and indexed from a unique ID. CD8+T cell counts were analysed as mean averages as to account for samples with incomplete sets of ROIs. Parametric variance was inspected by one-way analysis of variance with Tukey’s HSD post hoc using the Benjamini Hochberg (FDR) correction. Where data was determined to be non-parametric, Kruskal-Wallis with post hoc Dunn test, Sidak and Benjamini-Hochberg was employed as appropriate.

## Results

The confirmed LS cohort from BOLT numbered 60 ECs. These were combined with ECs from PORTEC 1 and 2, which had all undergone prior molecular classification. In total 706 ECs were stained, however, 99 tumours had either a scanning or ROI acquisition failure, meaning 607 ECs contributed to the analysis. The study schema is outlined in online supplemental figure S2. Overall, 2303 ROI were analysed, of these 328 (15%) had to be re-scored manually due to cell phenotyping inaccuracies, namely non-CD8+T cells (eg, erythrocytes) being incorrectly counted as CD8+T cells. The demographics of the analysed EC are outlined in table 1. Tumours were predominantly low stage due to the inclusion criteria of the PORTEC trials.

**Table 1:** Demographic data of tumours by molecular group

	LMB (n=413)	Conf' Lynch (n=50)	MLH1.meth (n=96)	path_POLE (n=23)	Lynch like (n=25)
Age at diagnosis (SEM)	67.5 (2.5)	53.1 (1.7)	65 (2.2)	63 (2)	64 (2.7)
Histology					
Endometrioid	401 (97%)	45 (90%)	95 (99%)	22 (98%)	24 (96%)
Clear cell	2 (0.5%)	0	0	0	0
Undifferentiated	1 (0.2%)	3 (6%)	1 (1%)	1 (2%)	0
Serous	8 (2%)	0	0	0	1 (4%)
Mixed epithelial	1 (0.2%)	2 (4%)	0	0	0
Grade					
1	297 (72%)	26 (52%)	29 (30%)	15 (67%)	18 (71%)
2	54 (13%)	14 (28%)	35 (37%)	2 (7%)	6 (25%)
3	62 (15%)	10 (20%)	32 (33%)	6 (26%)	1 (4%)
Stage					
I	397 (96%)	48 (96%)	95 (99%)	23 (100%)	25 (100%)
II	16 (4%)	1 (2%)	1 (1%)	0	0
III	0	1 (2%)	0	0	0
IV	0	0	0	0	0

Conf', confirmed; LMB, low mutational burden; Meth', methylation.

It was not possible to acquire all four ROI in all tumours. All samples from the LMB, path_*POLE*, Lynch Like and *MLH1*-methylation cohorts were cut from hysterectomy slides. In the confirmed LS cohort, 7 samples were cut from endometrial biopsies with no tumour/myometrial interface and therefore only two ROI could be generated within the tumour core. In several hysterectomy derived samples, it was not possible to secure four ROIs due to insufficient tumour bulk, regions of poor fixation or tumour that was confined to the endometrial cavity. In the LMB cohort 56 tumours had less than four ROI (one ROI n=2, two ROI n=23, three ROI n=31). For the Lynch Like cohort there were three samples with less than four ROI (3 ROI n=3), *MLH1*-methylation group four samples were less than four ROI (two ROI n=3, three ROI n=1), path_*POLE* group four samples were less than four ROI (one ROI n=1, three ROI n=3) and in the confirmed LS group 17 were less than four ROI (two ROI n=7, three ROI n=10).

**Figure 2:**
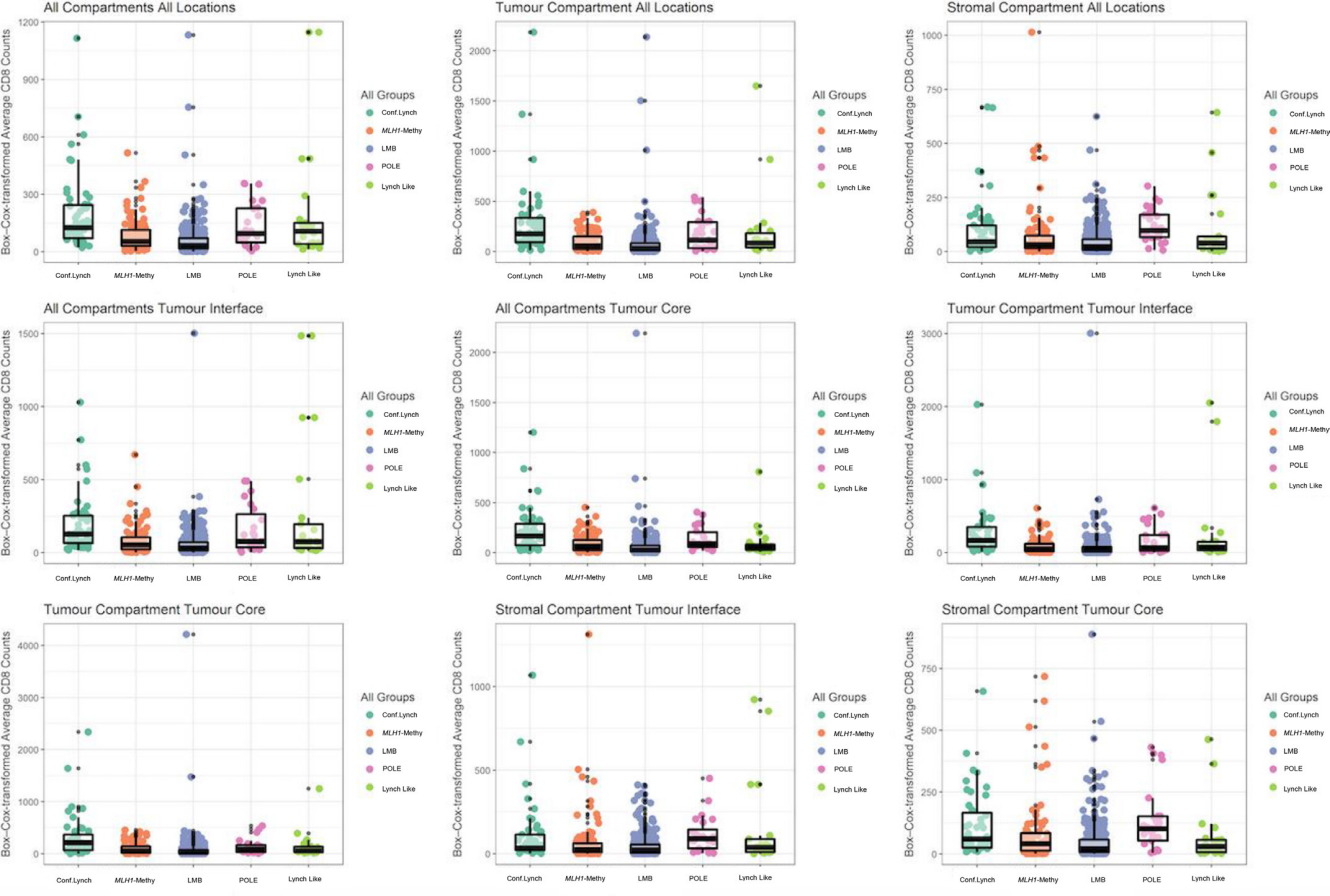
Variation in CD8+T cell counts across various tumour compartments as categorised by endometrial cancer molecular subtype. Conf, confirmed; LMB, low mutational burden; MLH1-Methy, hypermethylation of the promotor region of MLH1.

**Figure 3:**
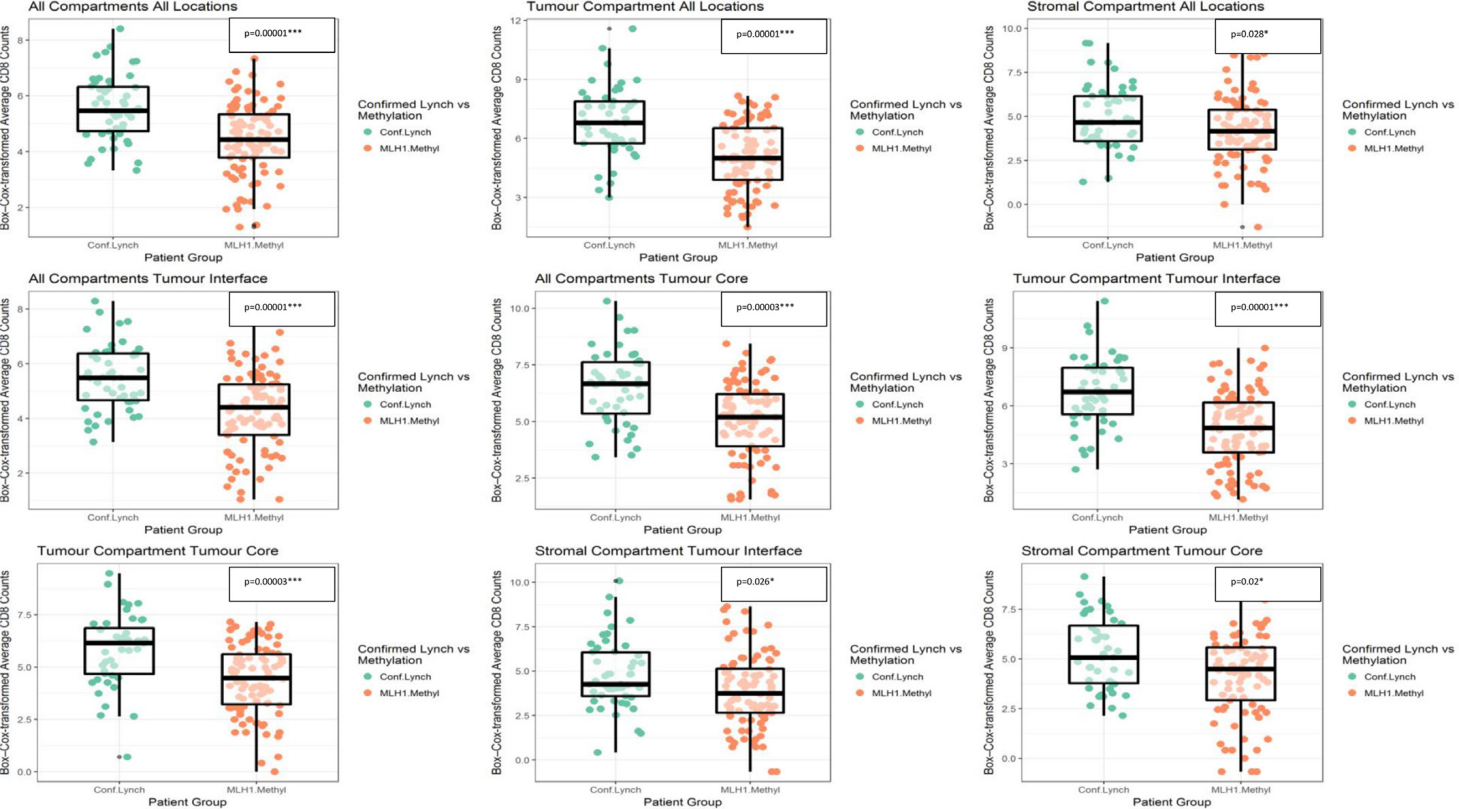
Box and whisker plots comparing the CD8+ counts of confirmed Lynch syndrome endometrial cancers versus MLH1_methylated endometrial cancers in various tumour locations. Conf, confirmed; MLH1 Methy, hypermethylation of the promotor region of MLH1.

Comparative statistics are sumerised in table 2 and figure 2. CD8+T cell counts in confirmed LS ECs were around threefold higher than in the *MLH1*-methylated cohort, excluding the tumour stroma. Confirmed LS, Lynch Like, *MLH1*-mehtylated and path_*POLE* ECs had significantly higher CD8+T cell counts across all tumour locations when compared with LMB. This difference was less pronounced in CD8+T counts between path_*POLE* versus confirmed LS ECs. Interestingly, CD8+T cell counts were significantly higher in the tumour compartments of confirmed LS ECs when compared with those of the Lynch Like group. However minimal differences were observed across all locations between the Lynch Like versus *MLH1*-methylated ECs (figure 3). Looking at all Lynch (confirmed LS+Lynch Like) ECs versus *MLH1*-methylated, all had significantly higher CD8+T cell count averages in all compartments excluding stromal compartment within the tumour core (online supplemental figure S3 and table S2), however, this result was weighted by the confirmed LS cohort. When all MMRd (confirmed LS+Lynch Like+*MLH1*-methylated) versus path_*POLE* ECs were analysed, path_*POLE* ECs had significantly higher CD8+T cell count averages in the stromal compartments (online supplemental figure S4). When looking at variation in CD8+T cell counts within LS itself, there was no significant difference between the average stomal or tumour CD8+T cell counts as per the gene affected (online supplemental figures S5 and S6). However, it should be noted that this analysis was impacted by small numbers.

**Table 2:** Direction and degree of significance in CD8+T cell counts between different molecular cohorts

	n1	n2	Direction of association	Significance
Conf. Lynch vs MLH1-Methy				
Overall	73 073	63 563	Conf. Lynch>MLH1-Methy	0.00001*
Tumour (all)	53 514	35 817	Conf. Lynch>MLH1-Methy	0.00001*
Stroma (all)	19 559	27 746	Conf. Lynch>MLH1-Methy	0.028†
Tumour interface (all)	37 900	31 652	Conf. Lynch>MLH1-Methy	0.00001*
Tumour core (all)	35 173	31 911	Conf. Lynch>MLH1-Methy	0.00003*
Tumour interface (tumour)	27 198	17 696	Conf. Lynch>MLH1-Methy	0.00001*
Tumour core (tumour)	26 316	18 121	Conf. Lynch>MLH1-Methy	0.00003*
Tumour interface (stroma)	10 702	13 956	Conf. Lynch>MLH1-Methy	0.026†
Tumour core (stroma)	8857	13 790	Conf. Lynch>MLH1-Methy	0.02†
Conf. Lynch vs LMB				
Overall	73 073	164 602	Conf. Lynch>LMB	0.00001*
Tumour (all)	53 514	102 593	Conf. Lynch>LMB	0.00001*
Stroma (all)	19 559	62 009	Conf. Lynch>LMB	0.00001*
Tumour interface (all)	37 900	85 415	Conf. Lynch>LMB	0.00001*
Tumour core (all)	35 173	79 187	Conf. Lynch>LMB	0.00001*
Tumour interface (tumour)	27 198	54 059	Conf. Lynch>LMB	0.00001*
Tumour core (tumour)	26 316	48 534	Conf. Lynch>LMB	0.00001*
Tumour interface (stroma)	10 702	31 356	Conf. Lynch>LMB	0.0005‡
Tumour core (stroma)	8857	30 653	Conf. Lynch>LMB	0.00001*
POLE vs Conf. Lynch				
Overall	28 491	73 073	NA	0.13
Tumour (all)	16 650	53 514	NA	0.13
Stroma (all)	11 841	19 559	POLE>Conf. Lynch	0.035†
Tumour interface (all)	15 365	37 900	NA	0.091
Tumour core (all)	13 126	35 173	NA	0.196
Tumour interface (tumour)	9385	27 198	Conf. Lynch>POLE	0.04†
Tumour core (tumour)	7265	26 316	NA	0.063
Tumour interface (stroma)	5980	10 702	NA	0.13
Tumour core (stroma)	5861	8857	NA	0.27
POLE vs MLH1-Methy				
Overall	28 491	73 073	POLE>Conf Lynch	0.0397†
Tumour (all)	16 650	53 514	POLE>Conf Lynch	0.039†
Stroma (all)	11 841	19 559	POLE>Conf Lynch	0.0003‡
Tumour interface (all)	15 365	37 900	POLE>Conf Lynch	0.032†
Tumour core (all)	13 126	35 173	POLE>Conf Lynch	0.049†
Tumour interface (tumour)	9385	27 198	NA	0.06
Tumour core (tumour)	7265	26 316	NA	0.17
Tumour interface (stroma)	5980	10 702	POLE>Conf Lynch	0.0028‡
Tumour core (stroma)	5861	8857	POLE>Conf Lynch	0.009‡
POLE vs LMB				
Overall	28 491	164 602	POLE>LMB	0.00001*
Tumour (all)	16 650	102 593	POLE>LMB	0.00001*
Stroma (all)	11 841	62 009	POLE>LMB	0.00001*
Tumour interface (all)	15 365	85 415	POLE>LMB	0.00001*
Tumour core (all)	13 126	79 187	POLE>LMB	0.00001*
Tumour interface (tumour)	9385	54 059	POLE>LMB	0.0024‡
Tumour core (tumour)	7265	48 534	POLE>LMB	0.0017‡
Tumour interface (stroma)	5980	31 356	POLE>LMB	0.00001*
Tumour core (stroma)	5861	30 653	POLE>LMB	0.00001*
MLH1-Methy vs LMB				
Overall	63 563	164 602	MLH1-Methy>LMB	0.00001*
Tumour (all)	35 817	102 593	MLH1-Methy>LMB	0.00001*
Stroma (all)	27 746	62 009	MLH1-Methy>LMB	0.015†
Tumour interface (all)	31 652	85 415	MLH1-Methy>LMB	0.0041‡
Tumour core (all)	31 911	79 187	MLH1-Methy>LMB	0.00001*
Tumour interface (tumour)	17 696	54 059	MLH1-Methy>LMB	0.0096‡
Tumour core (tumour)	18 121	48 534	MLH1-Methy>LMB	0.00003*
Tumour interface (stroma)	13 956	31 356	NA	0.12
Tumour core (stroma)	13 790	30 653	MLH1-Methy>LMB	0.006‡

Significance shown as a p value as calculated by the Kruskal-Wallis rank sum test. The direction of association is expressed as X>Y whereby the CD8+T cell count would be significantly higher in X than it is in Y. If there was no significant difference between the CD8+T cell count the value ‘NA’ is shown.

* Very very significant.

† Significant.

‡ Very significant.

Conf. Lynch, confirmed Lynch syndrome carriers; LMB, low mutational burden; MLH1-Methy, endometrial cancers with hypermethylation of the MLH1 promotor region; POLE, DNA Polymerase Epsilon, Catalytic Subunit.

## Discussion

This study reports a comprehensive description of CD8+T cell distribution in ECs by molecular subtype. This work explored the application of automated cell counting as a means of high-volume TILs phenotyping. Our findings indicate that *MLH1*-methylated ECs are immunologically distinct from ECs in confirmed LS carriers. Furthermore, Lynch Like ECs, a group that will include both somatic and inherited MMRd, are not immunologically comparable ECs found in women with confirmed LS. Finally, path_*POLE* ECs are as immunogenic, if not more so, than LS associated ECs indicating that they may be ideal targets for immunotherapy. These data could have significant implications for the application of immune treatments in ECs and our understanding of the immune environment of EC.

LS carriers are known to survive multiple, normally lethal, primary cancers.^28 29^ This is despite cancers in LS being associated with locally aggressive and poor prognostic features when compared with MMR proficient cancers.^14^ This may be explained by the association between MMRd ECs and high numbers of TILs; a phenomenon that is well established.^11^ Furthermore, higher numbers of TILs are associated with better survival.^13 17^ MMRd therefore seems to generate an immune response which in turn confers a survival benefit for the patient. The accumulation of frame shift mutations that occur in a MMRd cancer leads to the production of frame-shift peptides, de facto neo-antigens, which in turn elicit a tumour specific immune response.^18^ This mechanism is also seen in path_*POLE* cancers.^30^ Such immunogenic cancers invoke immune-editing and escape mechanisms often via upregulation of the PD-1/PD-L1 pathway.^10^ However, PD-1 and PD-L1 expression have been found to be poor proxies of immunotherapy response.^31^ Furthermore, recent murine data has indicated that somatically driven MMRd leads to heterogenous immunological response in part due to the timing of MMR inactivation and the variation in clonal expansion.^32^

Case reports have described favourable treatment outcomes from the application of PD-1 blockade in path_*POLE* cancers.^15 33^ Furthermore, recent trials have explored the application of PD-1 blockade in MMRd cancers, reporting positive results.^20 21 34 35^ The best treatment response was seen in those tumours with the highest CD8+T cell numbers; specifically, high CD8+T cell densities (cells/mm^2^) within the tumour core. However, traditionally trials heavily rely on cancers in LS patients with up to 48% carrying germline path_*MMR*.^20 34^ This does not reflect clinical practice where the majority of MMRd cancers arise from somatic hyper-methylation of the *MLH1* promotor region; this is especially the case in EC.^22 36^ No subgroup analysis was performed in these studies exploring the differences in response between somatic versus germline derived MMR loss as it was assumed their immune response was homogenous. However, there are distinct differences between these groups.^11 13^ Furthermore, not all MMRd ECs respond to immunotherapy, and therefore the use of CD8+T cell density could be a means to better stratify treatments.^17 21^ Certainly, the variation in treatment response that has been noted in clinical trials can potentially be explained by the variation in MMRd aetiology.^35^ Our results confirm that CD8+T cell numbers is significantly different between LS confirmed and *MLH1*-methylated ECs.

This observation is in keeping with the literature exploring the biology of these tumours. LS carriers have a defective MMR gene from conception. Although they carry a wild type allele, which for the most part preserves MMR system function, the mutant allele is expressed and this halplosufficiency may lead to low levels of MSI.^37^ In addition, they are more likely to periodically develop MMRd cells which do not develop into a malignancy.^38^ These two mechanisms explain the presence of a frame-shift peptide specific immunity in healthy LS carriers.^39^ Therefore, LS carriers develop a pre-primed immune system with a memory of MMRd generated frame-shift peptides, so that when a MMRd EC develops, they mount a more pronounced and cytotoxic immune response. As somatic hypermethylation of the promotor region of *MLH1* is an acute event, that is not present from conception, the period of haplosufficiency is less than in LS. In addition, the change is clonal and not constitutional. Therefore, the degree of frame-shift peptide production is likely to be lower than in LS healthy carriers as far fewer cells are affected in the pre-cancerous stage. This may limit priming of the immune system and reduce the magnitude of a subsequent immune response to sporadic EC.^32^

Another finding was that path_*POLE* somatic ECs have a similar CD8+T cell count to LS ECs. *POLE* is vital in DNA synthesis and maintaining DNA fidelity.^30^ Path_*POLE* ECs have an ultra-mutated phenotype with a mutational burden that exceeds MMRd-EC.^7^*POLE* ultramutated-ECs produce a rich abundance of antigenic neoepitopes compared with MMRd cancers.^40^ Furthermore, path_*POLE* are an early event in sporadic cancers and therefore the production of neoantigens is early and persistent during tumourigenesis.^40^ Due to this massive and sustained immune activation, it follows the immune response seen in path_*POLE* ECs would be similar to that seen in LS ECs, where neo-antigens are less abundant but produced over many years. Interestingly we did not see a variation in CD8+T cell counts within LS itself, in that no specific gene (path_*MLH1*, path_*MSH2*, path_*MSH6* or path_*PMS2*) demonstrated a significant difference in CD8+T cell density. This is expected given the dimeric relationship seen within in MMR proteins in which if one is defunct its partner protein is also often absent. Therefore, a defective MMR gene leads to global consequences with the MMR system.^41^

Our study has several key strengths. First, it includes over 600 ECs, giving a broad spectrum of samples from which, we derive our analysis. Second, the molecular groups were defined using diagnostic methods used clinically, and therefore reflect real world experience. We included a large cohort of ECs arising in confirmed LS carriers; to the authors’ knowledge, this is the largest number of such samples to undergo CD8+T cell phenotyping. Third, all our automated tissue segregation and counts were reviewed manually to ensure accuracy. Finally, this paper explores a crucial clinical question: are LS ECs immunologically different to other MMRd ECs? Our data suggesting they are, could inform better selection of EC patients for immunotherapy and this phenomenon should now be explored in treated cohorts.

Our study has limitations. Many of the ECs were sourced from the prospectively recruited PORTEC 1 and 2 studies, which restricted trial entry to stage 1 (PORTEC 1) and high-risk low stage ECs (PORTEC 2). It is unclear whether our findings are applicable to advanced stage ECs where immunotherapy arguably has most clinical utility and application. Women recruited to the BOLT study who contributed to the confirmed LS cohort were mostly LS ECs survivors; this could mean the cohort represented ECs with a survivable biology. However, LS ECs are known to have an excellent prognosis, more so than sporadic disease and therefore we believe these samples remain clinically representative.^42^ Furthermore, due to the design of our study, we cannot explain why we see a difference in CD8+T cells here. We do not know whether this is due to increased cellular recruitment/chemotaxis, cell survival or clonal expansion. In addition, due to the volume of samples analysed in this study, we did not phenotype the subsets of CD8+T cells or other immune cells within our samples. Of note, there is a considerable difference in age between those with *MLH1*-methylated ECs (mean age 65 years old at diagnosis) and those with confirmed LS (53 years old at diagnosis). This is expected due to the inherited nature of LS. However, it means that we cannot exclude the possibility that age is a confounding factor in our findings. Finally, our cohorts did not have meaningful representation of non-white women. This prevented us from exploring the impact of ethnicity on immune microenvironment.

In conclusion, to our knowledge, this work presents the most comprehensive phenotyping of CD8+T cells in ECs stratified by molecular subgroup. This study found that CD8+T cell counts and distribution are not equal between *MLH1*-methylated and confirmed LS ECs and we have outlined the potential biology underlying these differences. Our findings call for a review of current trial data looking to the application of immunotherapy in MMRd cancers. These trials have historically heavily relied on patients with LS. Our data may indicate this group is more susceptible to these treatments than the more clinically numerous, sporadic MMRd endometrial tumours. CD8+T cell density may be an important prognostic indicator of immunotherapy response and should be further explored in prospective treatment cohorts.

## Supplementary material

10.1136/bmjonc-2024-000320 online supplemental file 1

## Data Availability

Data are available upon reasonable request.
