# Supplementary material for: Patterns of cytotoxic T-cell densities in immunogenic endometrial cancers reveal a potential mechanism for differences in immunotherapy efficacy
Source: BMJ Oncol. 2024 May 21;3(1):e000320. doi: 10.1136/bmjonc-2024-000320 (PMC11235053; doi:10.1136/bmjonc-2024-000320)
Supplement: online supplemental file 1 [file bmjonc-2024-000320supp001.pdf]

CD8+ve cell density is distinct across molecular groups in endometrial cancer.

Supplementary Materials

Table of contents

Table S1 Details of the immunofluorescence immunohistochemistry panel components ______3

Figure S1: Data quality assurance workflow example ___________________________________4

Figure S2: Study flow schema ____5

Figure S3 All compartment analysis of CD8 counts between Any Lynch (Confirmed Lynch + Lynch Like) vs *MLH1*-Methylation ECs____6

Figure S4 All compartment analysis of CD8 counts between MMR deficient (Confirmed Lynch + Lynch Like+ MLH1-Methylation) vs path_*POLE* ECs _____________________________________7

Table S2 Raw count data (median average) of different tumour compartments between different molecular ____8

Figure S5 Stromal CD8 mean counts grouped by germline pathogenic variant carried. ________9

Figure S6 Tumour CD8 mean counts grouped by germline pathogenic variant carried. _______10

Target	Clone	Species	Isotype	Detection	Flurochrome	Supplier
Pan-Cytokeratin	AE1/AE3 and C11	Mouse	IgG1	Direct	Alexa 488	Thermo Fisher Scientific<br>Cell Signaling Technology
CD8	4B11	Rabbit	IgG2b	Direct	Alexa 594	Abcam
DAPI						

Table S1 Details of the immunofluorescence immunohistochemistry panel components

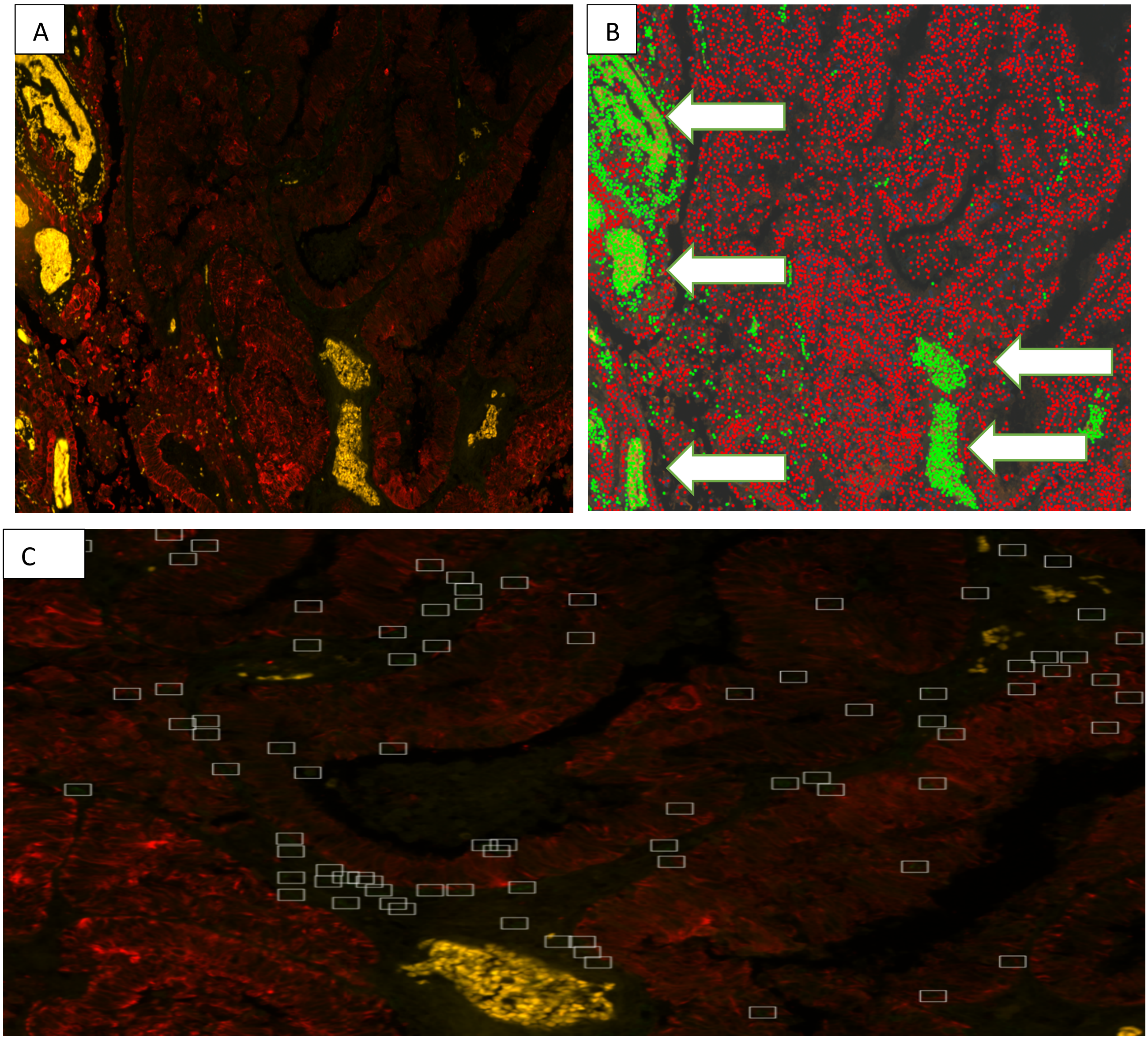

Figure S1: Data quality assurance workflow example. Composite image of sufficient quality (A), however cell phenotyping failure due to presence of erythrocytes being wrong phenotyped as CD8+ve cells (B) as denoted by arrows. Therefore, manual counting of CD8+ve cells (C) as denoted by boxes.

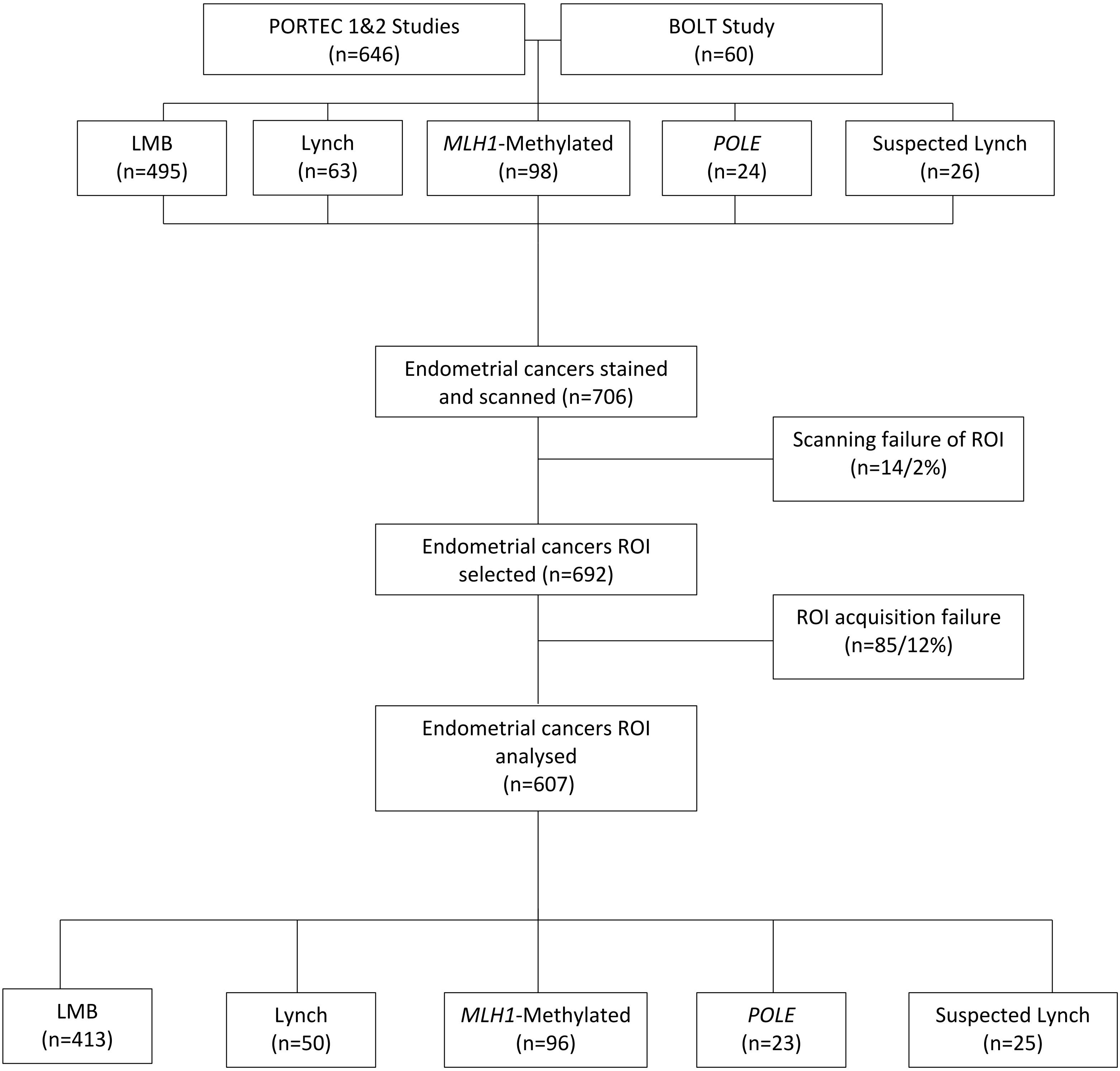

Figure S2: Study flow schema Abbreviations: PORTEC: Post-Operative Radiation Therapy in Endometrial Carcinoma study; BOLT: Biomarkers of Lynch Tumours study; LMB: Low Mutational Burden group; POLE: DNA Polymerase Epsilon, Catalytic Subunit; ROI: regions of interest.

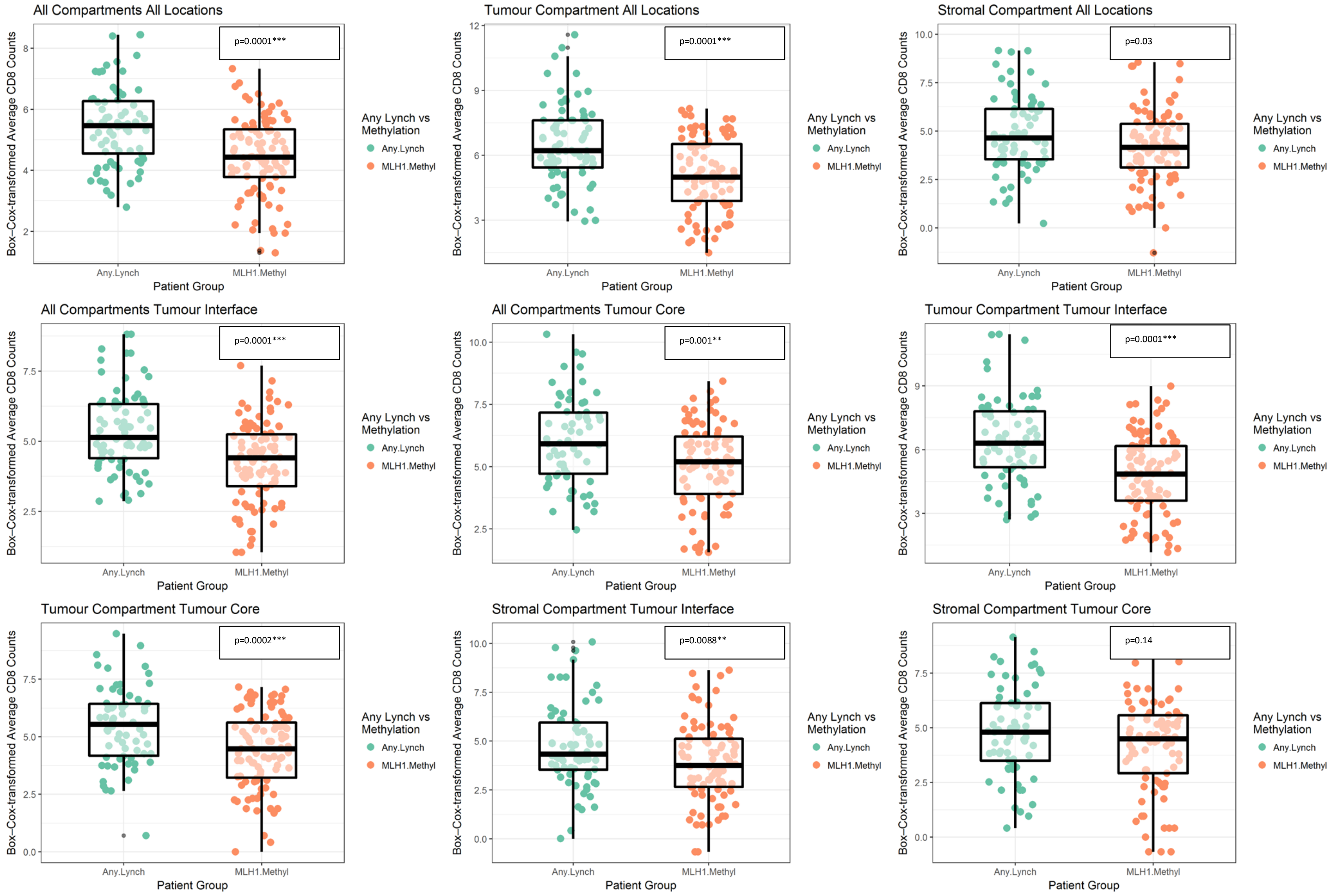

Figure S3 All compartment analysis of CD8 counts between Any Lynch (Confirmed Lynch + Lynch Like) vs *MLH1*-Methylation ECs.

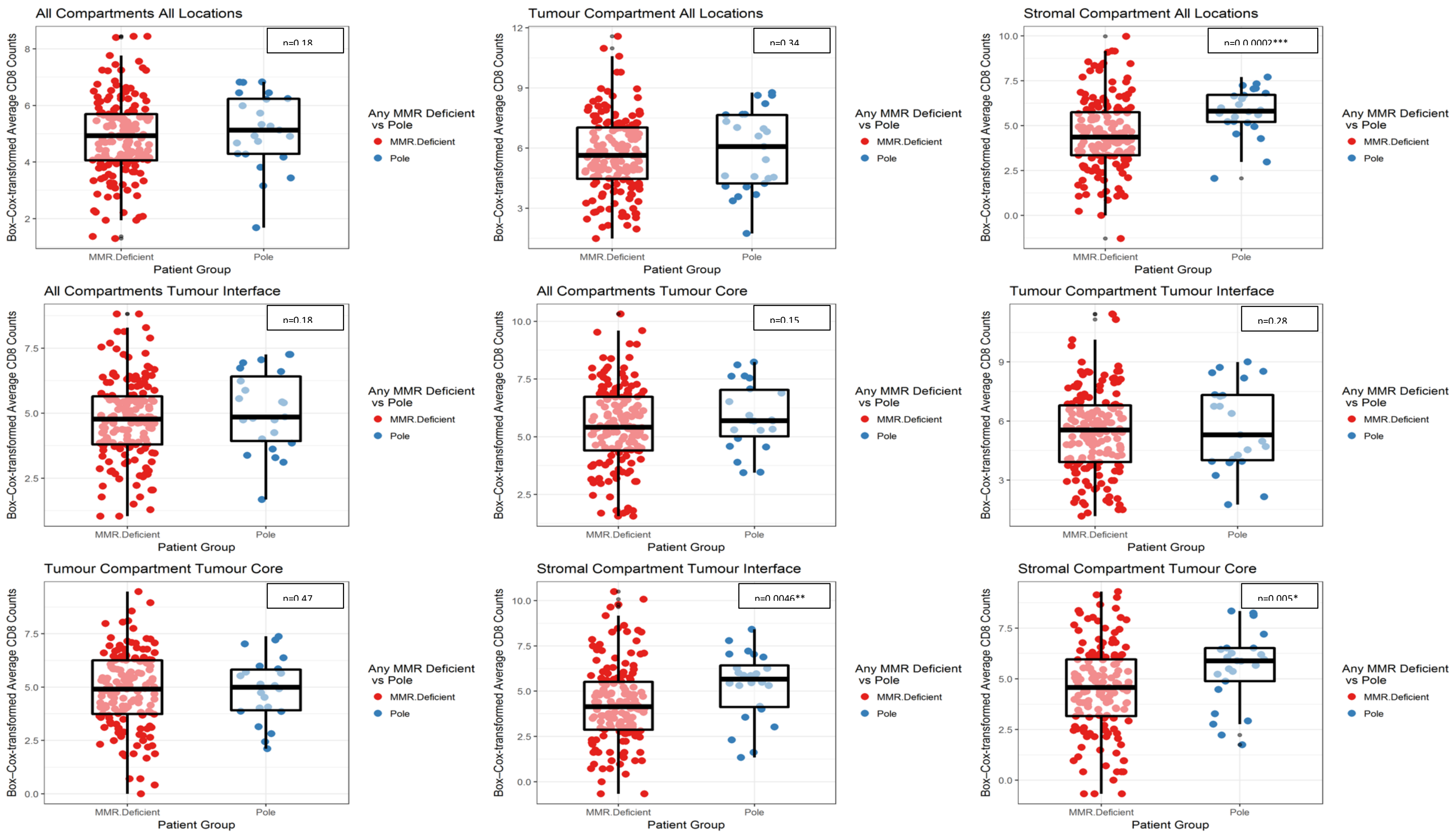

Figure S4 All compartment analysis of CD8 counts between MMR deficient (Confirmed Lynch + Lynch Like+ MLH1-Methylation) vs path_*POLE* ECs.

	Conf. Lynch	*path_POLE*	*MLH1-Methy*	*Lynch Like*	*LMB*
Overall	198.9 (21-1115)	96 (5-356)	55 (3-516)	107 (13-1146)	33 (1-1132)
Tumour (All)	178 (14-2184)	116 (5-542)	57 (4-389)	87 (13-1650)	36 (1-2137)
Stroma (All)	46 (3-668)	98 (7-302)	32 (1-1014)	40 (1-642)	23 (1-624)
Tumour Interface (ALL)	127 (18-1029)	77 (5-490)	53 (3-670)	77 (15-1485)	33 (1-1502)
Tumour Core (All)	166 (19-1200)	91 (19-405)	66 (4-452)	62 (9-806)	29 (1-2191)
Tumour Interface (Tumour)	170 (11-2028)	71 (5-612)	52 (3-609)	78 (12-2050)	34 (1-3003)
Tumour Core (Tumour)	215 (2-2340)	87 (8-534)	57 (1-455)	57 (15-1249)	31 (1-4213)
Tumour Interface (Stroma)	35 (2-1067)	90 (4-450)	24 (1-1311)	39 (1-921)	23 (1-411))
Tumour Core (Stroma)	60 (7-658)	102 (5-431)	41 (1-717)	30 (2-463)	21 (1-888)

Table S2 Raw count data (median average) of different tumour compartments between different molecular

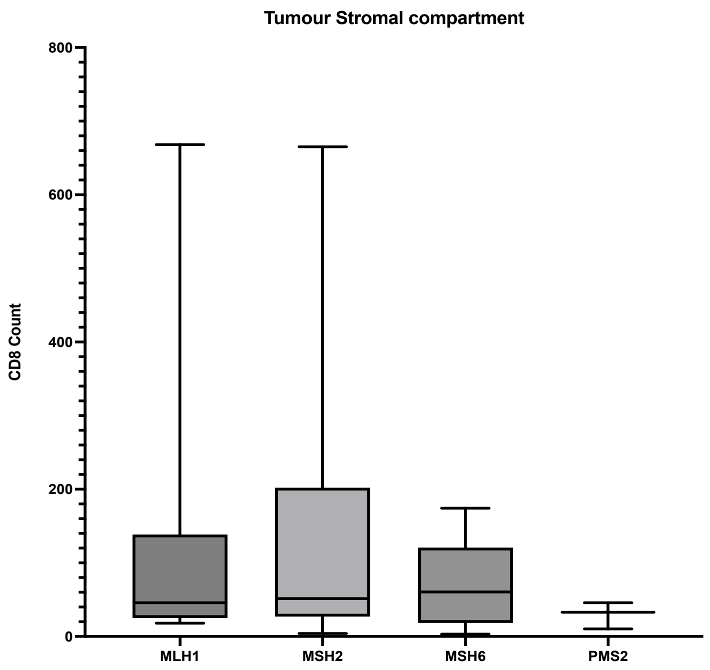

Table Analyzed	Stroma
Kruskal-Wallis test	
P value	0.5124
Exact or approximate P value?	Approximate
P value summary	ns
Do the medians vary signif. (P < 0.05)?	No
Number of groups	4
Kruskal-Wallis statistic	2.300
Data summary	
Number of treatments (columns)	4
Number of values (total)	49

	MLH1	*MSH2*	*MSH6*	*PMS2*
**Number of values**	11	19	16	3
**Mean**	124.4	142.6	74.39	29.75
**Std. Deviation**	189.2	173.3	58.97	18.1
**Std. Error of Mean**	57.05	39.77	14.74	10.45

Figure S5 Stromal CD8 mean counts grouped by germline pathogenic variant carried.

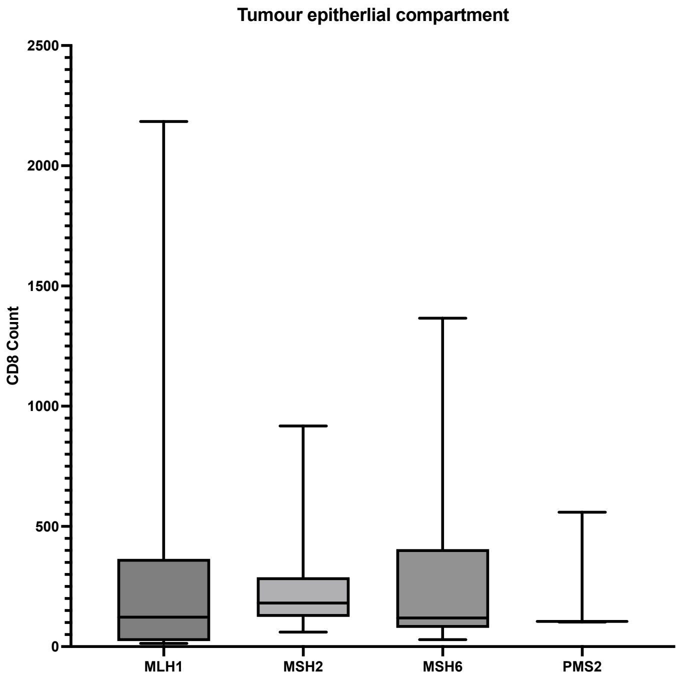

Table Analyzed	Tumour
Kruskal-Wallis test	
P value	0.7541
Exact or approximate P value?	Approximate
P value summary	ns
Do the medians vary signif. (P < 0.05)?	No
Number of groups	4
Kruskal-Wallis statistic	1.195
Data summary	
Number of treatments (columns)	4
Number of values (total)	49

	MLH1	*MSH2*	*MSH6*	*PMS2*
**Number of values**	11	19	16	3
**Mean**	362.4	263.3	276.8	255.4
**Std. Deviation**	624.3	208.8	339.4	262.9
**Std. Error of Mean**	188.2	47.9	84.85	151.8

Figure S6 Tumour CD8 mean counts grouped by germline pathogenic variant carried.
